# Study on the Dynamic Evolution Behavior and Failure Mechanism of Burn-Through Instability during In-Service Welding by Combining In-Situ Observation and Failure Analysis

**DOI:** 10.3390/ma16031184

**Published:** 2023-01-30

**Authors:** Hongjie Zhang, Tao Han, Yong Wang

**Affiliations:** School of Materials Science and Engineering, China University of Petroleum, Qingdao 266580, China

**Keywords:** burn-through instability, evolution behavior, crack, pinhole, chemical elements

## Abstract

There remains a lack of systematic understanding of burn-through instability, which also restricts the development of evaluation criteria. Based on the designed test device, the dynamic evolution behavior and failure mechanism of burn-through instability were investigated by combining in-situ CCD observation and failure analysis. In the initial stage of burn-through instability, the penetrating defect initiated beneath the molten pool bulge and propagated toward the arc. Finally, the weld centerline cracks or pinholes contributed to the pipeline failure. Based on in-situ observation, the burn-through pinhole was found to be forming in the overheating zone. Cracks and pinholes were found simultaneously in the burn-through instability zone and played an essential role in the burn-through instability. It could be concluded that a major burn-through hole was mainly developed from the fusion line to the inner wall surface along the wall thickness. According to the failure behavior and phenomena, the in-service burn-through instability area was divided into four parts, which were the damage failure (DF) zone, burn-through instability (BTI) zone, propagation (P) zone, and secondary burn-through zone (BT2). The failure mechanisms of the damage failure (DF) zone and burn-through instability (BTI) zone were significantly affected by the high temperature and plastic strain. The failure behavior of the propagation (P) zone was influenced considerably by the DF and BTI zones. The secondary burn-through zone was mainly affected by the high temperature. The uneven distribution of chemical elements showed an important influence on defect initiation.

## 1. Introduction

Pipeline risk control and safety reform have become the critical direction of the industry. Traditional pipeline repair (or rerouting) methods must stop and clean the conveying medium before the pipeline repair, which is bound to induce environmental pollution and economic loss. In-service welding repair refers to the method of directly welding the auxiliary pipe (such as the sleeve or branch pipe) to the in-service pipeline to repair the damaged pipelines [1,2]. It ensures the continuous operation of pipelines and is an environmentally-friendly, economical, and efficient repair technology for oil and gas pipelines. Due to the pipeline’s forced constraint and the flowing high-pressure medium, in-service welding repair has two main technical difficulties [3]. First is the risk of burn-though. In the process of in-service welding, the arc heat will make the pipe wall lose the ability to contain the internal pressure and allow the conveying medium to escape [4]. Second, the flow medium in the pipeline will increase the possibility of cold cracks in the weld and the heat-affected zone, leading to hydrogen-induced cracking. However, burn-through is the primary problem to be solved [5].

Because of the engineering application value, previous studies mainly focused on the burn-through criteria. Since the 1970s [6], burn-through evaluation methods have been continually improved. These evaluation methods can be divided into experimental approaches, thermal analysis models, and thermal-mechanical models.

1. Experimental approach

The early studies were conducted under specific working conditions. When the working conditions changed, the research results would show poor universality. According to API 1104 [7], the minimum safe wall thickness of the pipe was 6.4 mm when a low hydrogen electrode was used to conduct in-service welding. Burn-through was usually unlikely to occur when the pipe wall thickness was greater than 6.4 mm. This rule of thumb has good engineering application value and was widely accepted. With the application of high-strength pipeline steel in Australia [8], the wall thickness of the X70 and X80 pipes can be 3–5 mm. It has also been reported that some companies had successfully controlled the risk of burn-through instability when the wall thickness was reduced to 3.2 mm. However, there was still a lack of burn-through criteria when the pipe wall thickness was less than 6.4 mm.

2. Thermal analysis models

With the development of numerical simulation technology [9,10,11], the maximum inner wall temperature criterion based on the thermal analysis model was presented. Although experimental methods often provided relatively accurate results, the numerical simulation could significantly reduce research costs and address a broader range of in-service welding applications. In the late 1970s and early 1980s, BMI (Battelle Memorial Institute) developed a famous and fundamental thermal analysis model (Battelle model) and found that the burn-through was not likely to occur when the inner surface temperature did not exceed 982 °C (1800 °F) [12,13]. Later, PRCI founded the EWI (Edison Welding Institute) and developed a similar thermal analysis model (PRCI model) [14]. After that, another thermal analysis model (Equity Engineering model) was presented based on the Battelle model.

3. Thermal-mechanical models

The burn-through instability during in-service welding occurred with the interactions of welding multi-physics fields and pipe pressure. It was apparent that the thermal models did not consider the stress effect. Therefore, the thermal-mechanical models, which mainly included the residual strength model [15], radial deformation model [16], and plastic failure model [17], were proposed successively. The residual strength model, also known as the “cavity” model, incorporated material strength into the model based on the thermal analysis model. The high temperature significantly reduced the material strength during in-service welding. Hence, the residual strength model converted the high-temperature material strength to the full material strength at room temperature, and the reduced thickness of the pipe wall was treated as a cavity. Then the burn-through risk could be evaluated based on the ASME B31G procedure [18]. In recent years, Xue [19], Li [20], Guo [21], and Wu [22] also made efforts to develop the residual strength model. Essentially, the residual strength model did not consider the effects of welding stress and pipe pressure. In a strict sense, it was a thermal analysis model rather than a thermal-mechanical model.

In contrast, radial deformation and plastic failure models have successfully considered the stress effect. The pipe wall beneath the weld pool would be partially yielding, and the inner surface would be bulging (radial deformation), under the interactions of pressure and multi-physical fields. The radial deformation was a typical burn-through sign, and the welds would be treated as unsafe when the radial deformation exceeded 0.4 mm. However, this limit value was obtained under certain conditions. Thus, it might not always be reliable when the experimental conditions have changed. The plastic failure model was slightly different from other thermal-mechanical models. In this model, the yield stress was considered, and it pointed out that the burn-through would occur when two-thirds of the pipe wall was in a yield state. However, during the in-service welding, the stress state was very complex, and little literature was reported to clarify the stress distribution and its evolution behaviors [23,24].

Based on the consideration of engineering application, the burn-through instability models (criteria) seem to be very conservative. Especially for thin-walled pipelines, it was not easy to control the inner wall temperature below 982 °C during in-service welding. Many experimental tests also demonstrated that burn-through would not occur until the inner surface temperature approached 1260 °C [14,25]. Although many burn-through evaluation models have been proposed, no unified burn-through instability criteria has been formed, especially for thin-walled pipelines. The burn-through instability was a highly complex problem and was affected by many factors. In general, these factors could be grouped into three categories: the wall thickness of the pipeline at the in-service welding area; the welding parameters (e.g., current, voltage, travel speed); and the operation conditions (e.g., flow, pressure). There were many phenomena during the dynamic process of in-service welding burn-through instability. These phenomena mainly included the melting of the pipe wall, the high temperature and the strength reduction, the radial deformation of the inner surface, the stress induced by welding and pipe pressure, the weld centerline cracks and pinhole in the burn-through area, and the carburized and eutectic iron layers forming on the inner surface. All the burn-through evaluation models (or evaluation criteria) were built based on these phenomena, which implied that it was essential to understand the phenomena, behavior, and mechanism of in-service welding burn-through instability. We still lack a systematic understanding of the burn-through instability, which also restricts the development of evaluation criteria. However, only limited literature has investigated the phenomena, behavior, and mechanism of in-service welding burn-through instability.

Boring et al. [26] evaluated the burn-through risk based on their test device. They concluded that excessive inner diameter (ID) surface bulging, the carburized and eutectic iron layer on the ID surface, were the precursor of burn-throug instability. It was found that the carburized layer formed at 1600 °F (871 °C) and eutectic iron at 2066 °F (1130 °C). Based on failure analysis, Wu et al. [27,28] investigated the distribution characteristics of burn-through pinholes and discussed the evolution behavior of burn-through instability. It was found that the pinholes were located at the rear of the molten pool. It was also found that there were some cracks that appeared near the burn-through pinholes, and these cracks were parallel to the welding direction. Similar experimental phenomena were also reported by Bruce and Kiefner [29]. However, the pinholes were found in the front end of the weld pool. In addition, the formation mechanism of weld centerline cracks was not clarified. Boring and Bruce [30] pointed out it would result in a burn-through pinhole when the hoop stress was lower than 33% of the yield strength, and the failure mechanism would change to a weld centerline crack when the hoop stress was larger than 33%. Cracks and pinholes were often found simultaneously, and the pinhole was regarded as the leading cause of the burn-through instability. However, the effect of the cracks was often ignored. It was also suspected that the cracks developed during the cooling process and showed no effect on the burn-through instability. The formation mechanism of radial deformation was also not clear, and the effect of welding stress and pipe pressure on burn-through was often ignored. In theory, the dynamic process of burn-through failure requires excessive high-temperature deformation, initiation and propagation of micro-cracks, and formation of macroscopic defects (cracks or burn-holes). Since the initiation and propagation of microcracks occur below the molten pool, they are difficult to find. In contrast, the formation process of the burn-through pinhole is easier to capture because a huge sound often accompanies the formation of burn-through pinholes, and many tube media will escape from the pinhole. The previous investigations are mainly based on failure analysis, and the in-situ experiments were never introduced. Although we are interested in the initiation and evolution behaviors of burn-through instability, we still lack a systematic analysis and understanding of that.

In this work, in-service welding on the pressurized pipe was carried out based on the designed test device, and the dynamic behaviors of burn-through instability were observed with the help of a CCD camera. After this, the failure analysis and numerical simulation were conducted to clarify the burn-through instability’s evolution behaviors and failure mechanism in detail.

## 2. Materials and Experimental Procedure

### 2.1. Experimental Equipment and Material

**Figure 1:**
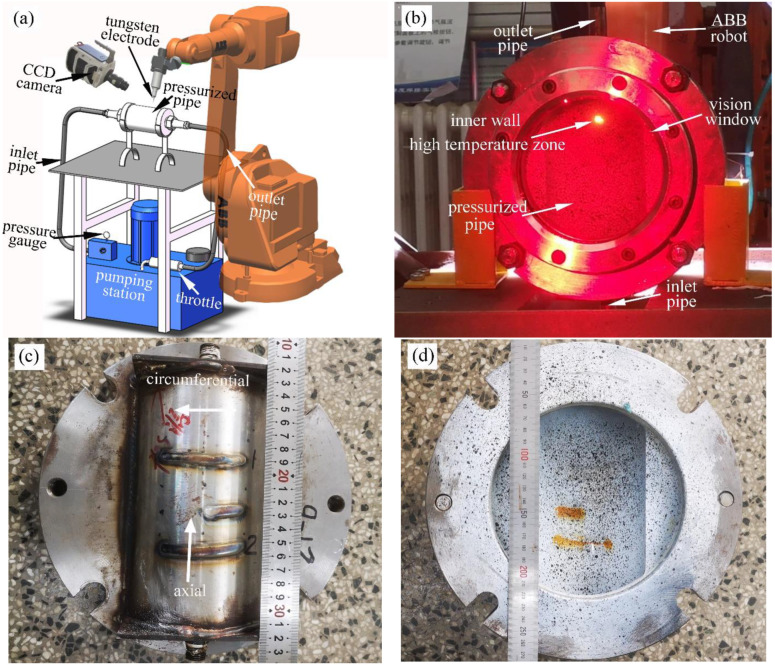
In-service welding device: (**a**) external observation device; (**b**) internal observation device; (**c**) top view of half-pipe set-up; (**d**) bottom view of half-pipe set-up.

In order to observe the dynamic behavior of the burn-through instability during in-service welding, two sets of test devices were designed in this paper, as shown in Figure 1. During in-service welding, the test device in Figure 1a could help observe the dynamic phenomena outside the pipe, while based on the test device in Figure 1b, the researcher could obtain the phenomena that occurred on the inner wall of the pipe. For simplicity, the test device shown in Figure 1a was named an external observation device, and the device in Figure 1b an internal observation device.

As shown in Figure 1a, the in-service welding system was composed of the pressurized pump station, pressure gauge, inlet pipe, X65 pipe, outlet pipe, and throttle. The pipe pressure (0–10 MPa) and water flow rate can be freely adjusted through the throttle valve. The flowing water was used as the medium to imitate the oil because it was much safer than oil. This method has been widely adopted in previous studies [31,32]. The first welding pass usually produced the highest burn-through risk during in-service welding. Hence, the investigations were all focused on the first welding pass. At present, automatic pipe welding technology has been gradually applied to in-service welding repair. In order to eliminate the influence of human factors and ensure the stability of the welding process, an ABB welding robot was used for the welding operation. TIG welding was used in this research. The tungsten electrode diameter is 3.2 mm, and the tip angle is 60°. During the welding process, the distance between the tungsten electrode tip and the outer surface of the pipe remained between 2–2.5 mm. The welding speed was set as 4 mm/s, and the thermal efficiency was taken as 0.70.

As shown in Figure 1b, the internal observation device was mainly composed of the inlet pipe, vision window, half-pipe set-up, and outlet pipe. In order to observe the inner surface phenomenon of the pipe, the pipe was cut into two half pipes along the axial direction and then made into the half-pipe set-up as shown in Figure 1c,d.

The material of the test pipe was X65 pipeline steel, with an outer diameter of 114 mm, a wall thickness of 4 mm, and a length of 200 mm. The chemical composition and mechanical properties of X65 pipeline steel are shown in Table 1 and Table 2, respectively.

### 2.2. In-Service Welding and Failure Analysis Process 

**Figure 2:**
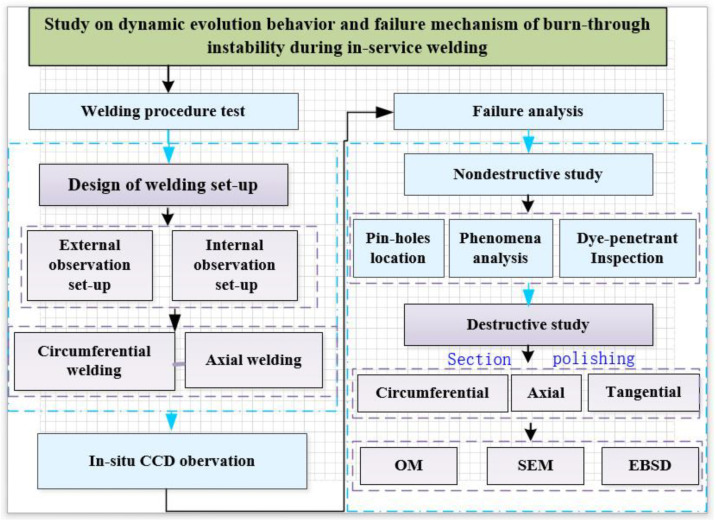
Flow chart of the experimental procedure.

The flow chart of the experimental procedure is given in Figure 2. Based on the developed test device, the pipe was welded in both circumferential and axial directions. The axial welding process was more affected by the medium pressure, and the welding area was less constrained. Hence, it was easy to produce large radial deformation and more conducive to observing the dynamic behavior of the burn-through instability. In the meantime, the in-service welding process could be observed based on the external and internal observation devices.

In previous studies, the medium pressure was generally controlled within a small (0–4 MPa) range to ensure the safety of the test. The pressure of the active pipeline was generally about 6–10 MPa, and the in-service welding was usually conducted under high pressure. Hence, the test pressures selected in this study were set as 3 MPa, 4.5 MPa, 6.3 MPa, 8.5 MPa, and 9.0 MPa, respectively. The welding current was gradually increased until the burn-through instability appeared, and all the burn-through instability tests were repeated more than two times under similar welding conditions. The circumferential in-service welding, observed with the external observation device, was conducted first, and the welding parameters can be found in Table 3.

Based on the internal observation device, the circumferential in-service welding test was conducted at the welding speed of 4 mm/s, and the test parameters are shown in Table 4.

Based on the external observation device, the axial in-service welding test was conducted at the welding speed of 4 mm/s, and the test parameters are shown in Table 5.

During the in-service welding process, a phantom VEO 1310L high-speed camera was used to observe the dynamic process of burn-through instability, and a CAVILUX^®^ HF high-speed camera laser lighting system was used to compensate for the light source. The acquisition resolution was 1280 × 960, and the sample rate was 9000 fps.

After welding, non-destructive studies were first performed. The camera and optical microscope (Leica DM2500 M, Wetzlar, Germany) were used to analyze the macroscopic morphology of the burn-through instability area. XTOM 3D collected the 3D profile and dimensional information of this area. The penetration inspection was applied to detect the cracks. Then, the test samples were analyzed for destructive sampling and characterization. Sampling was performed in the cyclic (longitudinal), axial (transverse), and tangential directions. After sample cutting, hand grinding and mechanical polishing were used to reduce the sample thickness layer by layer to look for typical failure defects such as cracks and holes.

## 3. Finite Element Modeling

**Figure 3:**
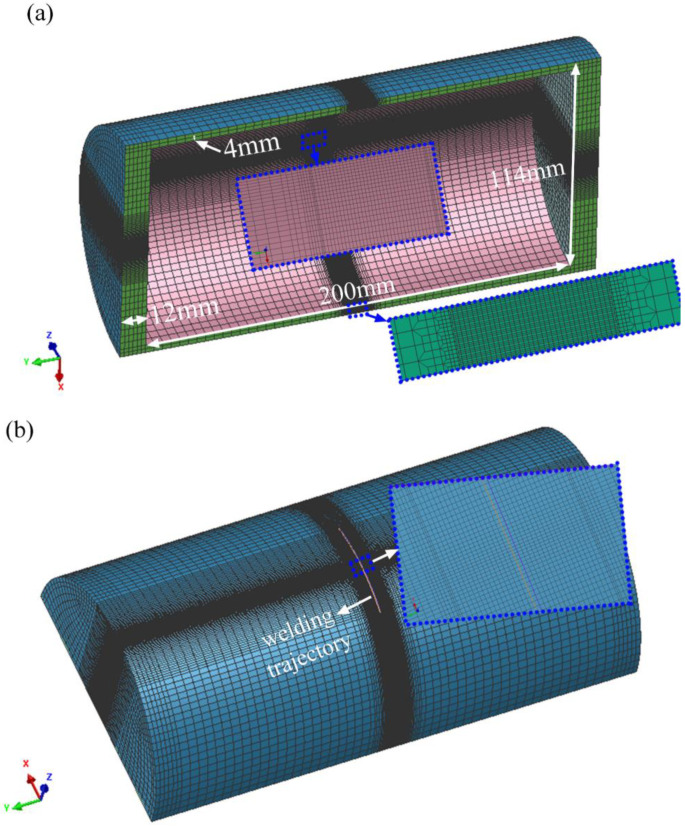
The half-pipe finite element model: (**a**) downside; (**b**) upside.

A sequentially-coupled thermal-metallurgical-mechanical (TMM) model was used to predict the temperature-stress field distributions. In order to reduce computational time, the 3D half-pipe finite element model was built according to the size of the experimental pipe, as shown in Figure 3. The smallest mesh size of the weld zone and HAZ (heat-affect zone) was set as 0.2 mm × 0.2 mm × 0.3 mm, and the element number was 224,848.

**Figure 4:**
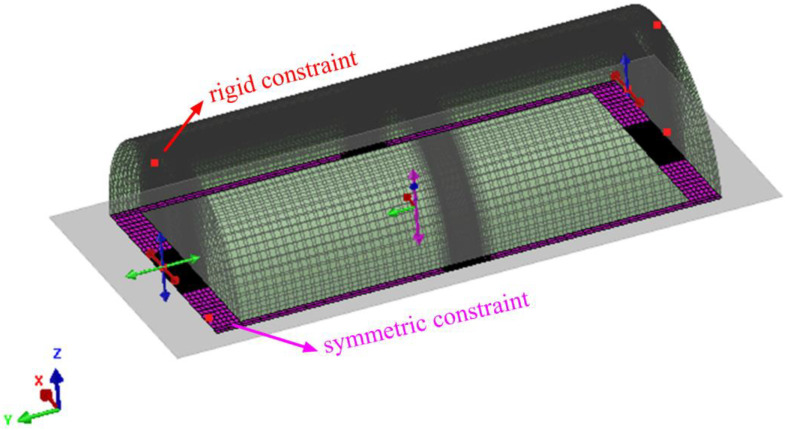
Restraint picture.

**Figure 5:**
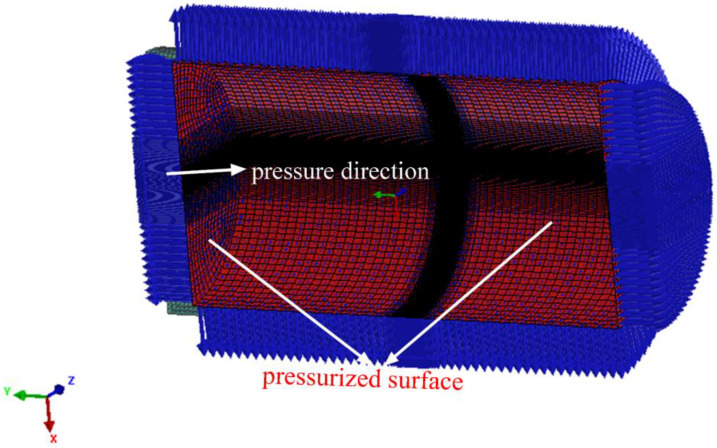
Pressure application.

The symmetric constraint was applied on the symmetry plane, and the rigid constraint was applied on the left and right planes, as shown in Figure 4. The pressure was applied as the experimental condition, as shown in Figure 5.

### 3.1. Thermal Analysis

The double ellipsoid heat source [33] was employed to describe the heat flux distribution of TIG, and the governing equations were given below:

(1) $$q_{f}(x,y,z)=\frac{12\sqrt{3}\eta UI}{(a_{f}+a_{r})b_{h}c_{h}\pi \sqrt{\pi }}\exp(-\frac{3x^{2}}{a_{f}^{2}}-\frac{3y^{2}}{b_{n}^{2}}-\frac{3z^{2}}{c_{h}^{2}}),x\geq 0$$

(2) $$q_{f}(x,y,z)=\frac{12\sqrt{3}\eta UI}{(a_{f}+a_{r})b_{h}c_{h}\pi \sqrt{\pi }}\exp(-\frac{3x^{2}}{a_{r}^{2}}-\frac{3y^{2}}{b_{n}^{2}}-\frac{3z^{2}}{c_{h}^{2}}),x\leq 0$$

where $q_{f}$ and $q_{r}$ are the power density functions (W m^−3^), η is the arc efficient, U is the arc voltage (V), I is the welding current (A), and $a_{f}$, $a_{r}$,$\;b_{h}$ and $c_{h}$ are the distribution parameters.

As reported in our previous study [34], the heat dissipation of the outer surface was treated as the natural convective heat transfer. The governing equation was expressed below:

(3) $$h_{outer}=-5.67\times 10^{-8}\times (T+T_{0})(T^{2}+T_{0}^{2})+h_{c}$$

where $T_{0}$ is the room temperature (K), and $h_{c}$(25 W m^−2^ K^−1^) is the convection coefficient.

The governing equation of forced convection heat dissipation for the inner surface is given below:

(4) hinner=0.8×5.67×10−8[(273.15+T0)+(273.15+T)]⋅[(273.15+T0)2+(273.15+T)2]+0.027ρfλfRef0.8Prf1/3μ432×e−T1831+1337×e−T270.14

where $\rho_{f}$ is the water density at the initial temperature, 999.9 kg/m^3^; $\lambda_{f}$ is the heat conductivity coefficient at the initial temperature, 55.1 W/(m•°C); μ is the kinematic viscosity coefficient, 1788 × 10^−6^ kg/(m•s); ${\mathrm{Pr}}_{f}$ is the Prandtl number; and Ref is the Reynolds number.

(5) $$P_{r}=\frac{\mu_{f}\cdot C_{pj}}{\lambda_{f}}$$

where $\mu_{f}$ is the kinematic viscosity coefficient at the initial temperature, 1788 × 10^−6^ kg/(m•s); $C_{pj}$ is the specific heat of the water at initial temperature, 4.212 kJ/(kg•°C); and $\lambda_{f}$ is the heat conductivity coefficient at the initial temperature, 55.1 W/(m•°C).

(6) Ref=Vf×ρf×Lxμf

where $V_{f}$ is the flow velocity of water, 0.041 m/h; $\rho_{f}$ is the water density at the initial temperature, 999.9 kg/m^3^; $L_{x}$ is the length of the test pipe, 200 mm; and $\mu_{f}$ is the kinematic viscosity coefficient at the initial temperature, 1788 ×10^−6^ kg/(m•s).

### 3.2. Metallurgical and Mechanical Analysis

The diffusion-controlled transformations (austenite-ferrite and the austenite-bainite transformations) [35,36] are described by the Johnson-Mehl-Avrami-Kolmogorov equation based on SYSWELD 2008, as given below:

(7) $$\frac{dP(T)}{\mathrm{dt}}=f(\overset{•}{T})\frac{P_{eq}-P(T)}{\tau (T)}$$

The shear-type transformation (austenite-martensite transformation) is described by the Koistinen-Marburger equation [24], as given below:

(8) $$P(T)=1-\exp[-b\times (M_{s}-T)]$$

where P is the martensite percentage, $M_{s}$ is the start temperature of martensite, and b(0.029) is the law parameter.

For mechanical analysis, the governing equation of the total strain is given below:

(9) $$\varepsilon_{total}=\varepsilon_{e}+\varepsilon_{p}+\varepsilon_{th}+\varepsilon_{tp}$$

where $\varepsilon_{e}$ and $\varepsilon_{p}$ are the elastic strain and plastic strain, respectively; and $\varepsilon_{th}$ and $\varepsilon_{tp}$ are the metallurgical strain and transformation-induced plastic strain, respectively.

**Figure 6:**
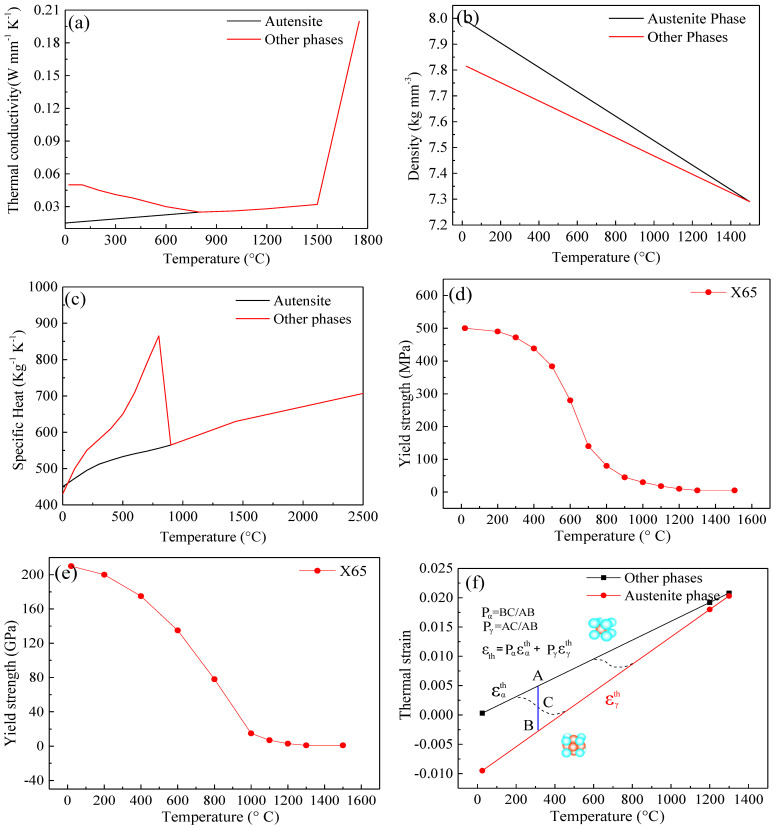
The material properties: (**a**) thermal conductivity; (**b**) density; (**c**) specific heat; (**d**) yield strength; (**e**) Young’s modulus; (**f**) thermal strain of Q345.

The isotropic hardening model and the von Mises criterion were employed to describe the yield and hardening behaviors during welding. Poisson’s ratio was set to be a constant of 0.33 during the numerical simulation. The material properties were given, as shown in Figure 6.

## 4. Results and Discussions

### 4.1. In-Situ Observation of In-Service Welding Burn-Through Instability

When the in-service welding was conduted on a large diameter pipeline, the high-temperature area can be regarded as a small pipe defect, and the stress will be redistributed in the weld joint. It is generally believed that the effect of the medium pressure on the burn-through instability is negligible. However, when the wall thickness is thin, the influence of the medium pressure will be significant. In essence, burn-through instability is a kind of high-temperature fracture. The dynamic process of burn-through instability consists of the initiation of micro-defects (cracks), crack propagation, and the formation of the macroscopic crack or burn-through pinhole.

The crack was usually developed parallel to the welding direction, and the stress that was perpendicular to the crack provided the driving force for crack propagation. During axial welding, the driving force was the hoop stress. During circumferential welding, the axial stress acted as the driving force for the crack. The axial welding process was more affected by the medium pressure, and the welding area was less constrained. Hence, it was easy to produce large radial deformation and more conducive to observing the dynamic behavior of the burn-through instability.

(10) $$\sigma_{\mathrm{hoop}}=\frac{PD}{2t}$$

(11) $$\sigma_{axial}=\frac{PD}{4t}$$

where $\sigma_{\mathrm{hoop}}$ and $\sigma_{axial}$ is the circumferential (hoop) and axial stress, respectively; P is the pipe pressure; D is the pipe diameter, and t is the wall thickness of the pipe.

The dynamic process of in-service welding was observed by a CCD camera, and the welding process parameters are shown in Table 6.

**Figure 7:**
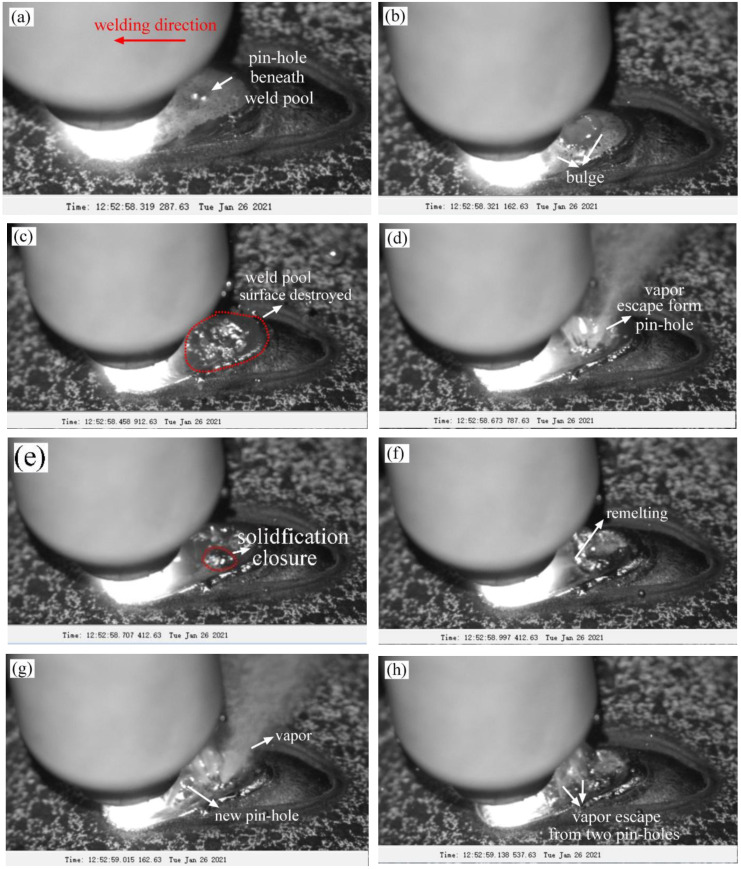

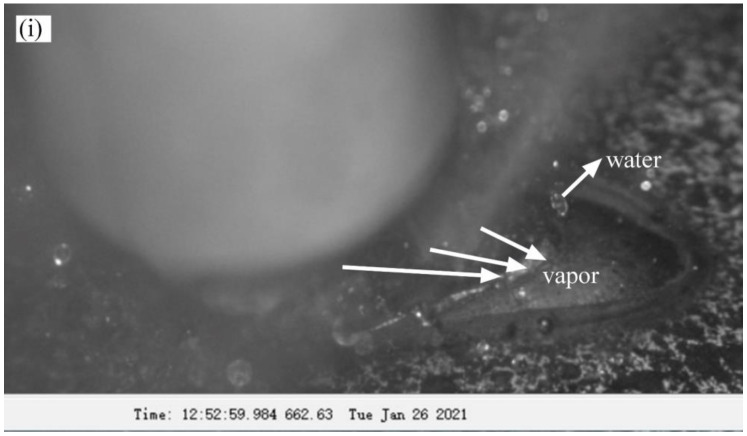
Dynamic evolution behavior during the burn-through process of the 26K-6: (**a**) 0.319 s; (**b**) 0.321 s; (**c**) 0.459 s; (**d**) 0.674 s; (**e**) 0.707 s; (**f**) 0.997 s; (**g**) 1.015 s; (**h**) 1.139 s; (**i**) 1.984 s.

The dynamic evolution behavior of the burn-through instability of 24K-6 was observed and analyzed in detail. As shown in Figure 7a, it could be observed that a bulging molten pool appeared behind the arc, and it was located at the end of the molten pool. The molten pool bulging implied that a penetrating defect (pinhole or crack) might have appeared under the weld pool at about 0.319 s. It also did not rule out that the excessive radial deformation caused the pool bulge. Here, we temporarily believed that the penetrating defect caused the molten pool bulge. At 0.321 s, the size of the penetrating defect (burn perforation or crack) became larger, and the radial deformation further increased. At 0.459 s, the defect below the melt pool further enlarged, and the vapor started to escape from the penetrating defect and destroyed the surface of the melt pool. The molten pool began to oscillate under the impact of the water vapor. At 0.674 s, the size of the penetrating defect continued to increase and developed into a larger burn-through hole. A large amount of vapor began to spray from it, and the molten pool was pushed to approach the welding arc. The arc stability was affected.

At 0.707 s, the molten pool continuously flowed backward and started covering the burn-through hole. Moreover, the weld pool beneath the burn-through hole began solidifying, and the solidified metal closed the hole. At 0.997 s, the new molten pool metal continued to flow back to the burn-through hole, and the hole was opened. As a result, a large amount of vapor escaped from the burn-through hole, the vapor took the molten metal away, and the thickness of the weld pool decreased. At 1.015 s, a new burn-through hole formed at the edge of the arc. When the size of the hole further increased, a larger amount of vapor was ejected from the hole, and the welding arc was extinguished.

**Figure 8:**
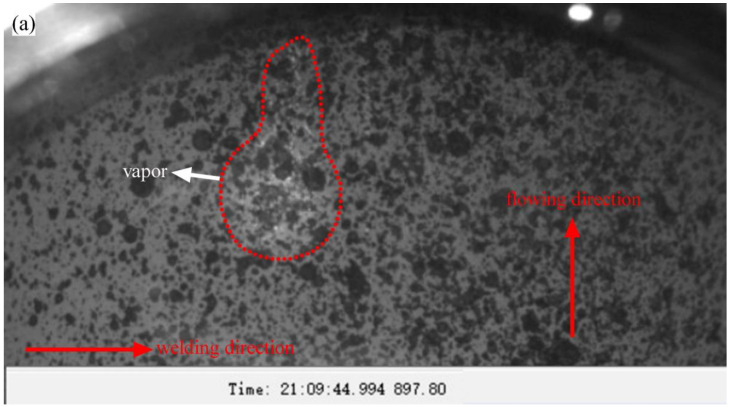

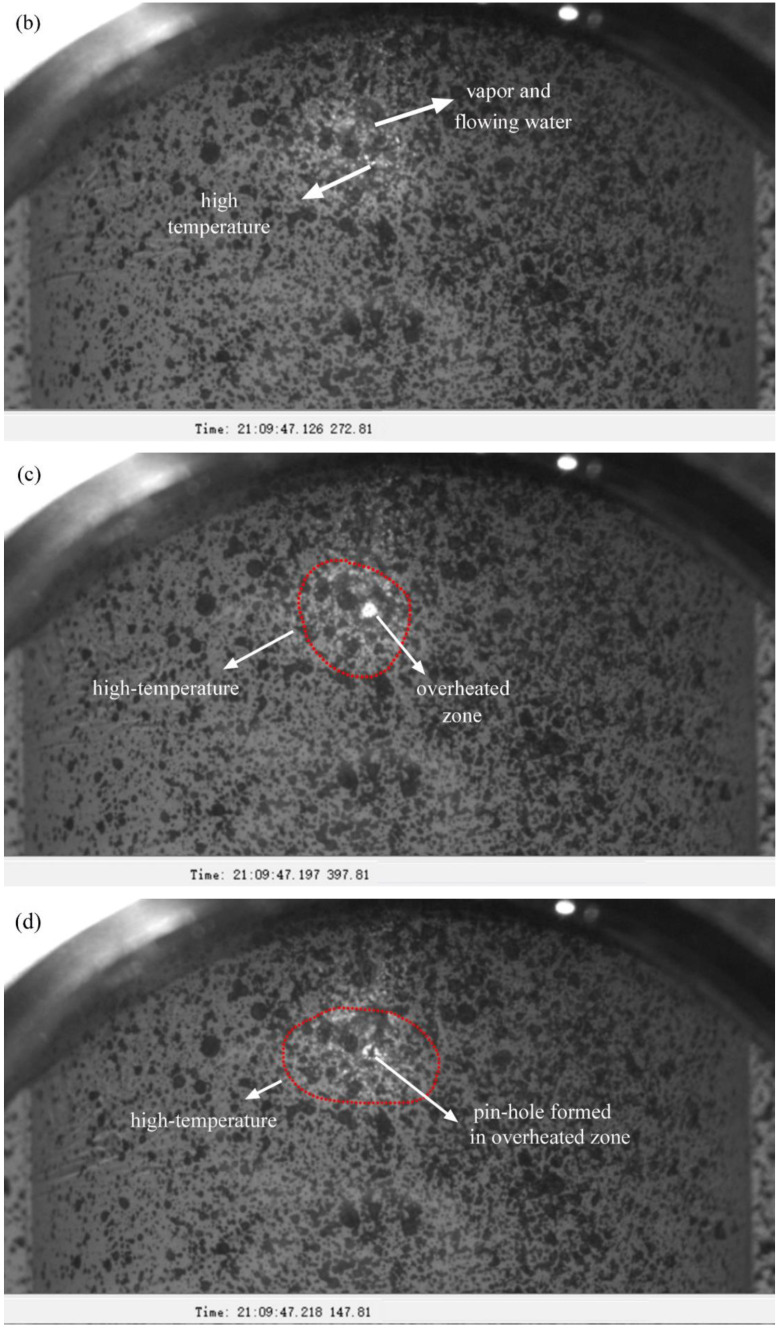
Dynamic evolution behavior during the burn-through process of the 26O-1: (**a**) 0.994 s; (**b**) 1.126 s; (**c**) 1.197 s; (**d**) 1.218 s.

Based on the external observation device, the dynamic behavior of the burn-through instability was observed in detail. However, all the phenomena occurred below the molten pool during the external observation, which was a relatively indirect observation. In order to obtain the phenomena on the inner surface of the pipe, the internal visualization device was introduced. As shown in Figure 8a, the welding direction was marked. The direction of water flow has been indicated in the figure. When the welding started, the temperature increased. The water was quickly heated to boiling point and produced a large number of bubbles. The pipe pressure increased by 0.3–0.6 MPa during this process. As shown in Figure 8b, the high-temperature area on the inner surface continued to become larger, and more bubbles were produced. As shown in Figure 8c, a high-temperature overheating area appeared on the inner surface. Then, a burn-through hole was formed in the overheating area, and a large amount of water began to be ejected. Even though the ABB welding robot was used, an overheating area might still be formed, which often developed into the initiation position of burn-through instability.

According to the analysis, axial welding made it easy to produce large radial deformation and more conducive to observing the dynamic behavior of the burn-through instability. Therefore, the 17-ZD-11 sample was selected for detailed analysis. The welding process parameters are shown in Table 7.

**Figure 9:**
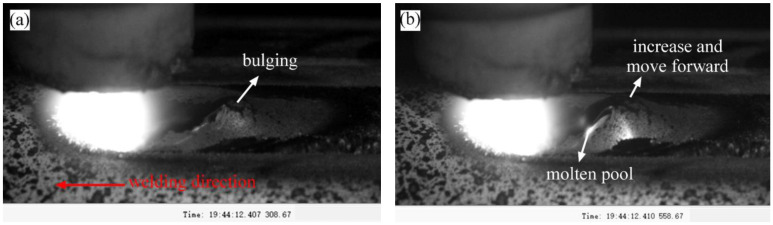

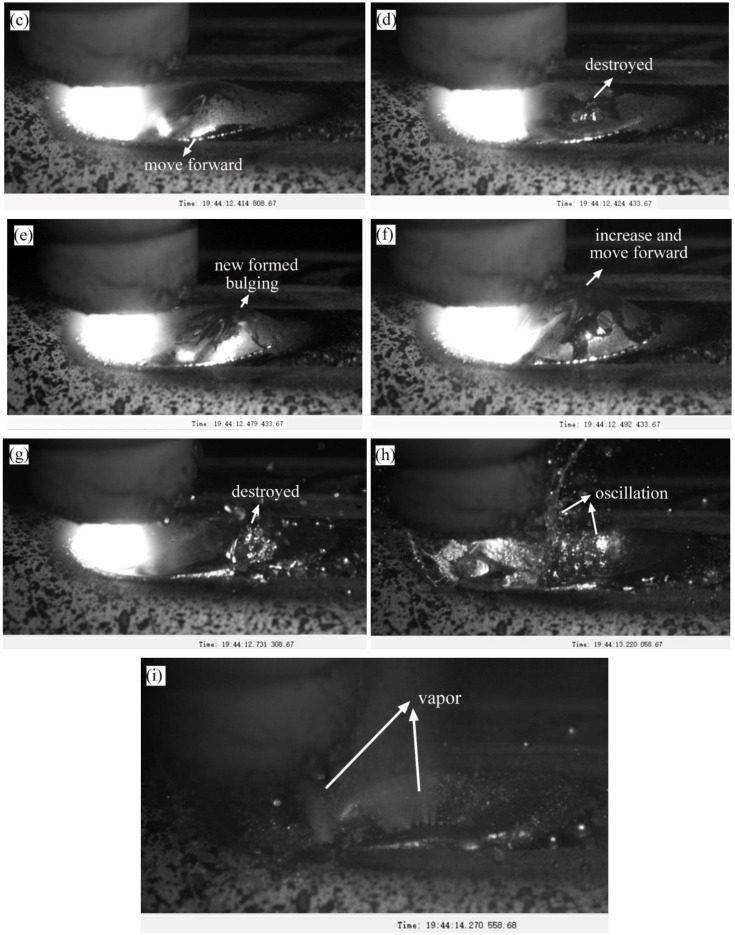
Dynamic evolution behavior during the burn-through process of the 17-ZD-11: (**a**) 0.407 s; (**b**) 0.411 s; (**c**) 0.415; (**d**) 0.424 s; (**e**) 0.479 s; (**f**) 0.492 s; (**g**) 0.731 s; (**h**) 1.220 s; (**i**) 1.271 s.

As shown in Figure 9a, molten pool bulging was also found in the early stage of burn-through instability (0.407 s). There are probably two potential explanations for this phenomenon. Firstly, it may have been produced by the instantaneous large radial deformation because the shape of the bulge was relatively smooth. The second possible reason is that it was caused by penetrating defects (pinhole or crack).

As shown in Figure 9b, at about 0.411 s, the size of the molten pool bulge increased and expanded rapidly. Then, the bulge propagated toward the arc and bulge morphology was destroyed when it touched the arc edge. After that, a new molten pool bulge appeared, and the dynamic evolution behavior described above repeated until the molten pool bulge ruptured, as shown in Figure 9g. As shown in Figure 9g–i, the vapor significantly affected the molten pool and arc stability. A large amount of vapor was then ejected from the penetrating defects.

Based on experimental results, the second reason was supported. Because the surface of the bulge ruptured in Figure 9d, the morphology was destroyed, and the size decreased rapidly, which indicated that a penetrating defect was formed under the molten pool. In addition, the dynamic evolution behavior repeated after that, which indicated that the bulge could not be caused only by the excessive radial deformation because it was unlikely to produce another similar radial deformation behavior in the same location.

As demonstrated by the experimental results, in the initial stage of burn-through instability, a large radial deformation formed beneath the bulge and penetrating defects (pinhole or crack) appeared. Then, the penetrating defects quickly propagated toward the arc. Finally, the weld centerline cracks or pinholes formed.

### 4.2. Macroscopic Morphology Analysis of the Burn-Through Instability Zone

**Figure 10:**
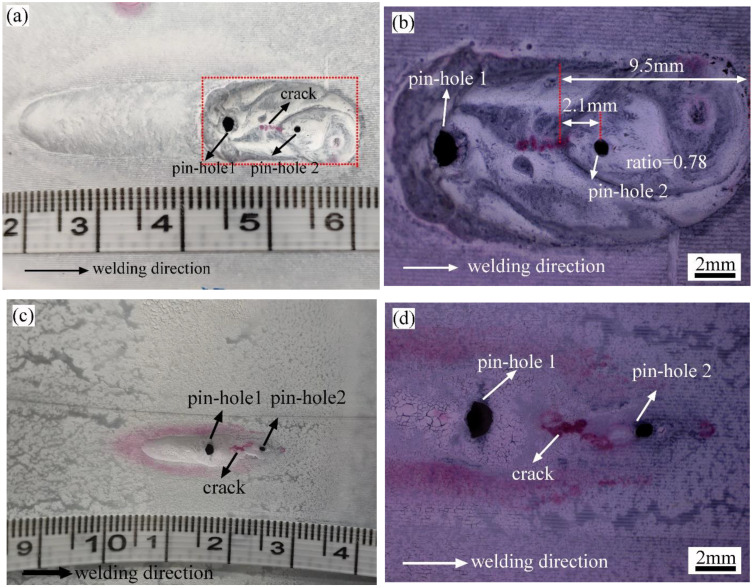
Macroscopic morphology and penetration inspection results near the burn-through zone: (**a**) upside; (**b**) detailed view of the burn-through zone; (**c**) backside; (**d**) detailed view of the burn-through zone.

After welding, the sample was cut off with a hand-grinding wheel and cleaned carefully with alcohol. A penetration inspection was applied to detect the cracks. The welding direction was marked in Figure 10a. It was found that there were two burn-through holes in the burn-through instability area. Pinhole 1 appeared before pinhole 2, and there was a weld centerline crack between the two pinholes. Both pinholes were located in the rear of the molten pool with an approximate ratio of 0.78. The weld centerline crack between two pinholes might be a penetrating crack, as demonstrated in Figure 10c,d.

**Figure 11:**
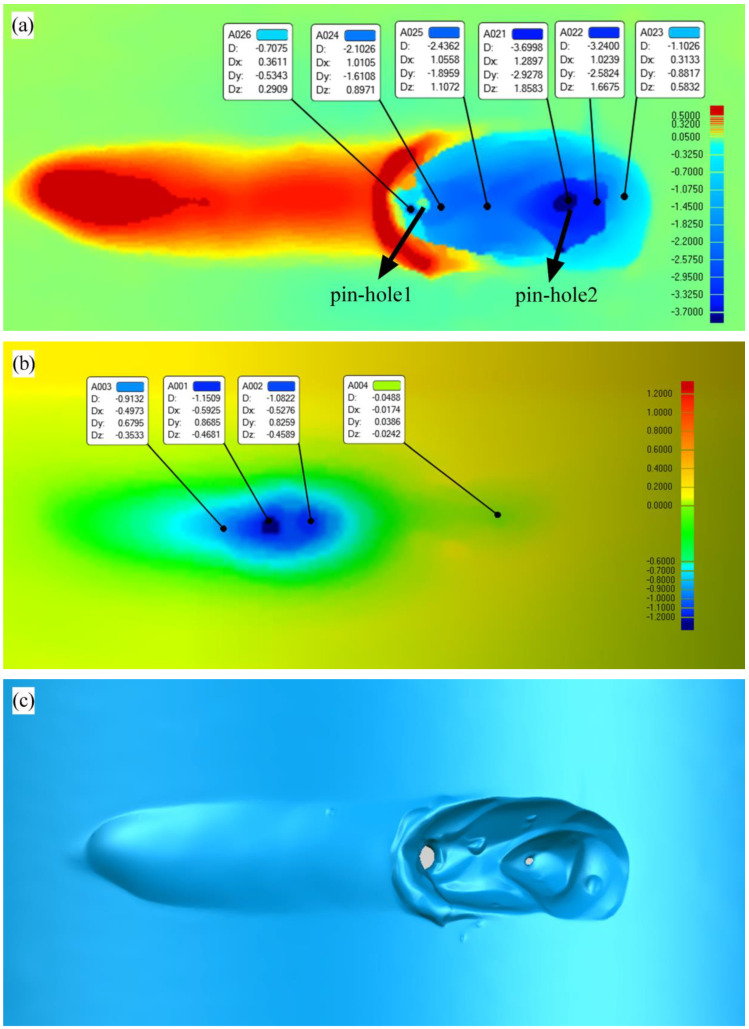

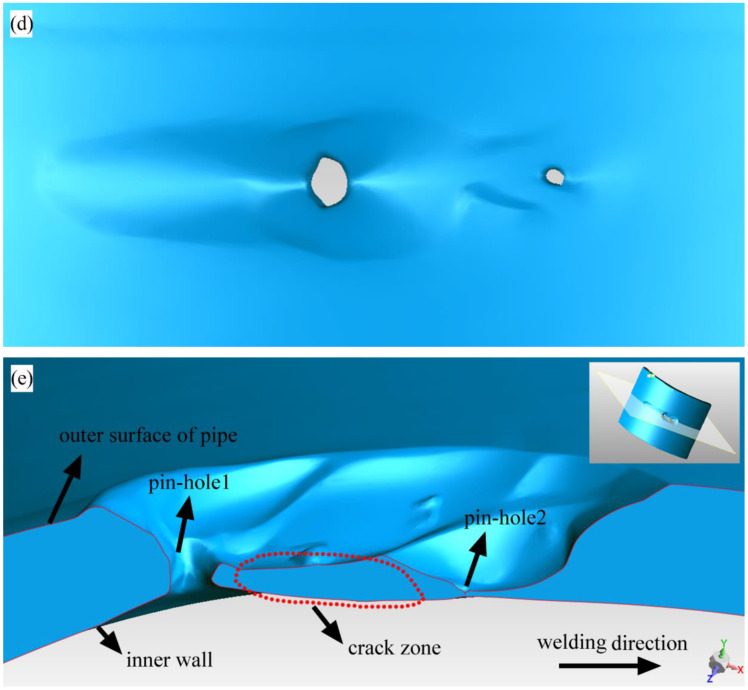
Macroscopic morphology and contours near the burn-through zone of 24E-1: (**a**) contour of the upside; (**b**) contour of the backside; (**c**) macroscopic morphology of the upside; (**d**) macroscopic morphology of the backside; (**e**) sectional view.

In addition, the 3D XTOM contour instrument was used to collect the morphology and size information of the burn-through instability zone. As shown in Figure 11a,c, most of the molten pool metal was removed by the ejected water. The maximum depth near pinhole 2 was about 3.7 mm, and the maximum depth near pinhole 1 was about 2.1 mm. In the dynamic process of burn-through instability, when pinhole 1 appeared, the arc was not extinguished, and the molten pool flowed backward from pinhole 2. Then, the molten pool metal solidified near pinhole 1. When pinhole 2 appeared, the arc was extinguished instantly, and a large amount of vapor escaped from the pinholes. It was found that an obvious local yield and radial deformation occurred near pinhole 1, while there was little radial deformation near pinhole 2. It demonstrated that the two pinholes obeyed different failure mechanisms. Pinhole 1 was more affected by the high-temperature deformation and stress, and pinhole 2 was more affected by the high temperature.

**Figure 12:**
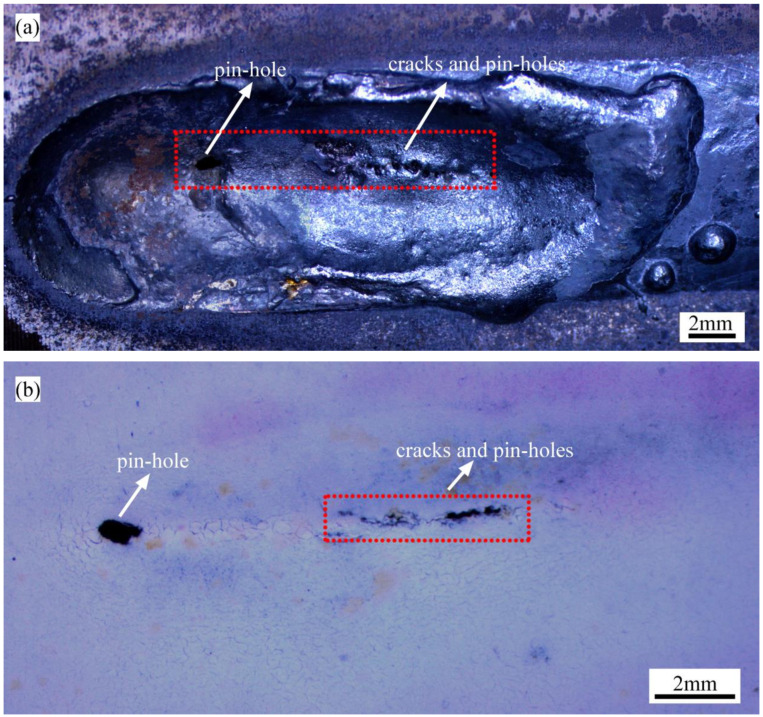
Macroscopic morphology near the burn-through zone of 17-ZD-11: (**a**) upside; (**b**) backside.

As shown in Figure 12, lots of pinholes and high-temperature cracks along the weld centerline were found in the 17-ZD-11 sample. As demonstrated in Figure 9, these weld centerline defects propagated rapidly after the appearance of the first pinhole. As a result, cracks often expanded along the weld centerline because of the higher temperature and bigger radial deformation in this position.

### 4.3. Mesoscopic Morphology Analysis of the Burn-Through Instability Zone

**Figure 13:**
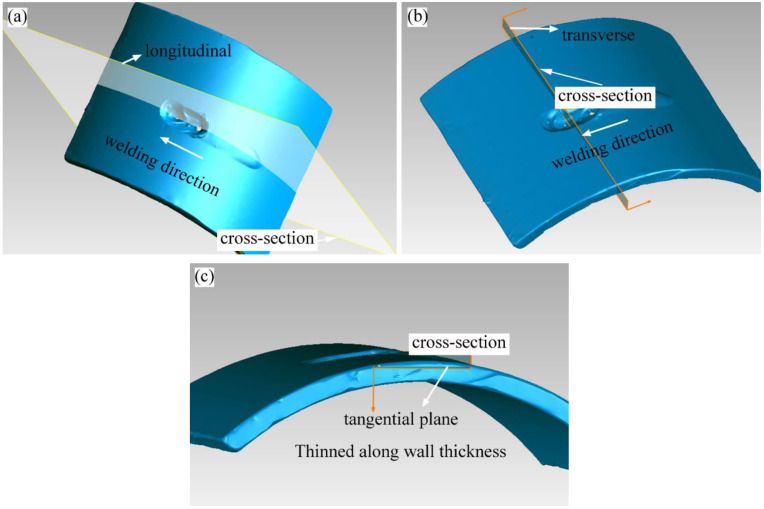
Method of cutting samples: (**a**) cut along the longitudinal path; (**b**) cut along the transverse path; (**c**) cut along the tangential path.

According to the definition method of welding residual stress, it was generally considered that the direction parallelled to the welding direction was the longitudinal direction, and the vertical direction was the transverse direction. The third direction was the tangential direction. The samples were cut in longitudinal, transverse and tangential directions. The schematic diagram is shown in Figure 13. After sampling, hand grinding and mechanical polishing were used to reduce the sample thickness layer by layer to look for typical failure defects. When the failure traces were found, the characterization instruments would further characterize the sample.

**Figure 14:**
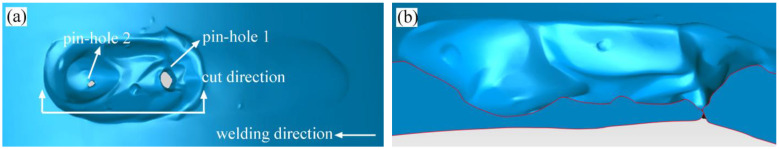

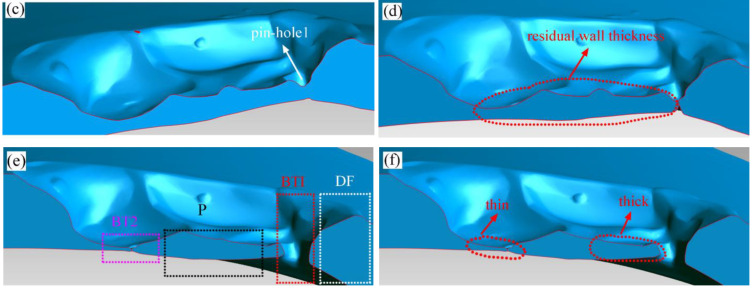
Thinning the sample layer by layer along the longitudinal direction. (**a**) cross section 1, (**b**) cross section 2, (**c**) cross section 3, (**d**) cross section 4, (**e**) cross section 5, (**f**) cross section 6.

The 24E-1 sample was used as a typical sample to analyze the evolution behavior of burn-through instability—cut the sample along the longitudinal direction near pinhole 1, and thin it layer by layer until the crack or defect was found. Figure 14b–f showed the topography of the sectional view during layer-by-layer thinning. According to the failure behavior and mechanism, the in-service burn-through instability area was divided into four parts. These were named the damage failure (DF) zone, burn-through instability (BTI) zone, propagation (P) zone, and secondary burn-through (BT2) zone, as shown in Figure 14e.

**Figure 15:**
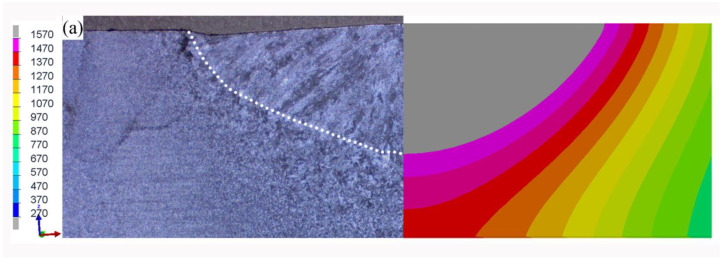

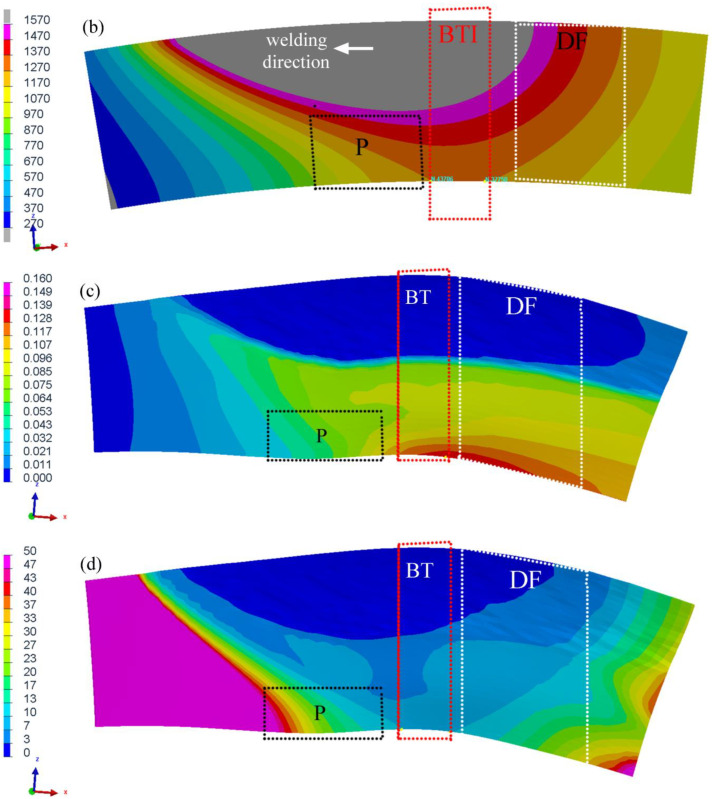

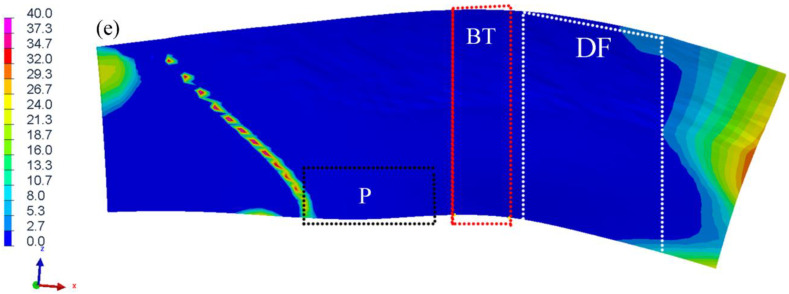
Simulation results of 24E-1: (**a**) experimental temperature distribution vs. the numerical result in the transverse section (°C); (**b**) temperature distribution in the longitudinal direction (°C); (**c**) plastic strain distribution in the longitudinal direction; (**d**) von Mises stress distribution in the longitudinal direction (MPa); (**e**) first principal stress distribution (MPa).

In order to further explain the dynamic behavior and mechanism of the burn-through instability, the distribution of the temperature field and strain field of 24E-1 were produced by SYSWELD. As given in Figure 15a, the experimental temperature distribution matched well with the predicted one, and the numerical simulation results showed good reliability. On this basis, the plastic strain contour, von Mises stress contour, and the first principal stress contour were listed.

According to Figure 15b, the burn-through instability (BTI) area produced the highest temperature and was more likely to lead to the burn-through hole. As shown in Figure 15c, it was also found that there was a bigger radial plastic deformation near the inner surface. Hence, the high-temperature crack was more likely to initiate in this location.

According to Figure 15b, the temperature in the damage failure area (DF) was slightly lower than that of the BTI area, but the inner surface of the DF area produced a larger plastic deformation. The first principal stress in this position is positive, so it was very easy to generate damage on the inner surface.

The temperature in the propagation zone (P) was slightly lower than that of the DF and BTI areas, and the plastic strain was also small. The weld centerline cracks were often found in this area. According to the numerical simulation results, it could be found that the crack driving energy of the P area was lower than that of the DF and BTI areas. Hence, the crack propagation needed to be driven by the penetrating defects formed in BTI and DF areas.

(1). The failure mechanism of the damage failure (DF) area

**Figure 16:**
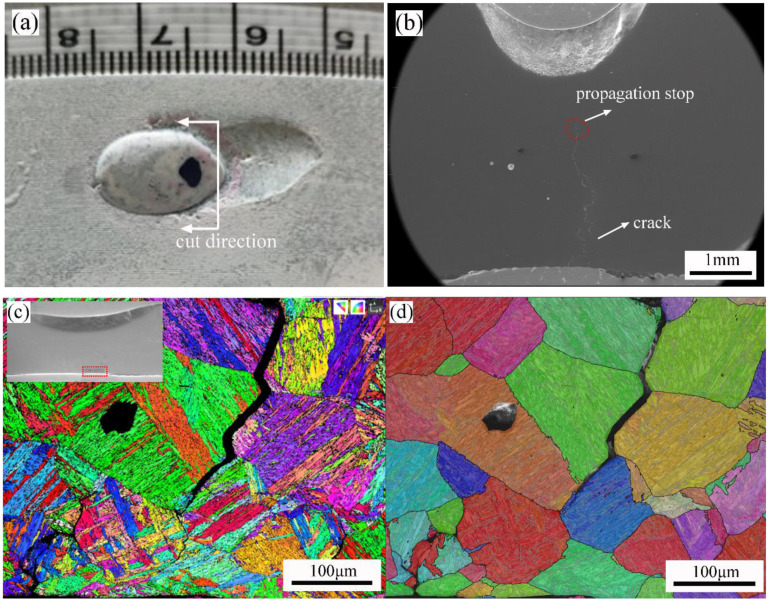
Failure behavior and mechanism analysis of DF zone: (**a**) schematic diagram of sample cutting; (**b**) morphology of crack; (**c**) IPF X; (**d**) prior austenite grain.

After cutting the specimen along the path shown in Figure 16a, the damage failure (DF) area morphology was obtained. Figure 16b showed that the crack did not penetrate the pipe wall. Instead, the crack seemed to have initiated from the inner surface and stopped in the middle of the pipe wall. According to Figure 15, the plastic strain near the inner surface was very large, and the first principal stress value was positive, which met the theoretical conditions of crack initiation. In contrast, the plastic strain started to get smaller in the middle of the pipe wall, and the crack was often easy to stop after expanding to this position.

According to Figure 15b, the temperature value of the plastic failure zone was above 1200 °C. The grain boundary strength was lower than the intragranular strength, so the crack was often developed along the prior austenite grain boundary.

It was found that prior austenite grain size (PAGS) significantly affected grain boundary sliding behavior, grain anisotropy, and intragranular/transgranular deformation behaviors, and thus also affected the crack behaviors. As shown in Figure 16d, when the PAGS was large, intragranular cracks tended to form near prior austenite grain boundary (PAGB). Transgranular cracking was found in the small prior austenite grains (PAGs) or the adjacent area of large and small PAGs. Based on the in-situ laser confocal tensile test at 1150 °C, it was found that small austenite grains grew rapidly by swallowing each other under the combined action of high temperature and load. During this process, the grain sizes of some PAGs did not get much bigger. Finally, grain uniformity was poor, as shown in Figure 16d. During the deformation process, deformed bands and twin boundaries appeared inside the large prior austenite grains, and the grain orientation was relatively hard. The internal deformation of small PAGs was mainly dominated by slipping, and the orientation was relatively soft. It was also reported [37] that the anisotropy was mainly determined by grain boundary sliding, and this anisotropy was attributed to the misorientation because it determined the decomposed shear stress along PAGBs. Refining of PAG size also helped reduce the deformation anisotropy and intergranular cracking. The maximum sliding distance distributed on a grain boundary of the large PAG also improved the possibility of intergranular cracking.

(2). The failure mechanism of the burn-through instability (BTI) area

**Figure 17:**
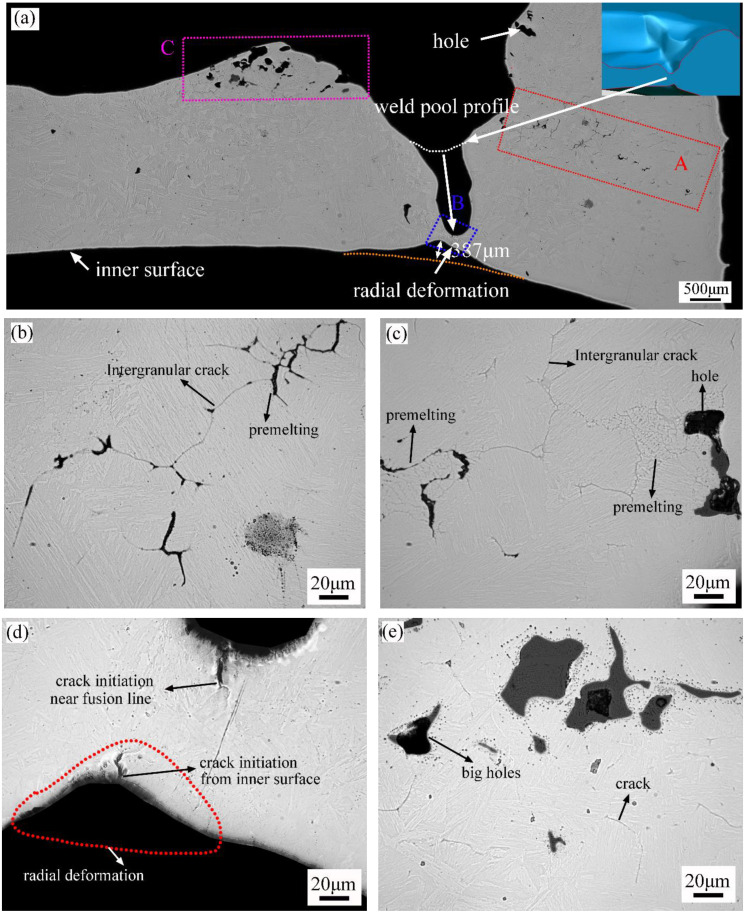
The morphology of the longitudinal section near burn-through hole 1: (**a**) global view of pinhole 1; (**b**) detailed view of location A; (**c**) detailed view of location A; (**d**) detailed view of location B; (**e**) detailed view of location C.

The longitudinal section diagram of 24E-1 was shown in Figure 17a, where the sample preparation process was between Figure 14c and Figure 14d. The metal of the pipe wall was melted on both sides of the molten pool. The melting trace was found near the welding pool profile. Area C in Figure 17a was located below the molten pool, and a large number of holes were formed in this area due to the high temperature. It was also found that there were a large number of high-temperature cracks near the burned hole. Area A in Figure 17a was located near the end of the molten pool, where numerous holes and cracks also existed. Figure 17b,c depict the detailed pictures of region A. A large number of pre-melting grain boundaries were found in this area [27,28].

The schematic diagram in the upper right corner of Figure 17a was obtained by XTOM reconstruction. By comparison, the depth of the melting pool further increased through the wall thickness, and a finger-like melting hole was formed. The residual wall thickness below was only about 150 μm. Combined with the results in Figure 8, it could be found that the temperature distribution below the molten pool was not uniform, and a sharp temperature increase would appear in a small area. High temperatures would lead to microscopic defects such as pre-melting, intergranular cracks and burned holes. These defects would act as the crack initiation source. When these microscopic defects connected and gradually expanded through the wall thickness, molten pool metal might further enlarge the defect size, and a finger-like hole would appear. The metal flowing rate would become slower when the weld pool was gradually eroded into the finger-like hole. Then, the temperature decreased, and a high-temperature crack would appear instead of the hole on the inner surface.

It could be concluded that a major burn-through hole was mainly developed from the fusion line to the inner wall surface along the wall thickness. The origin defects were often found below the fusion line, and the defects initiation was significantly affected by the temperature. However, the origin defects could initiate from many places. High-temperature defects would change into high-temperature cracks near the pipe’s inner surface because of excessive radial deformation.

**Figure 18:**
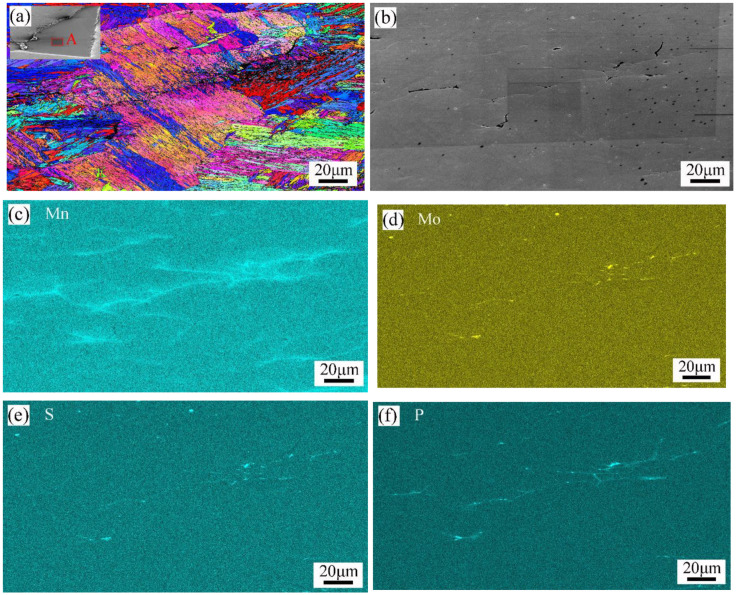
Microstructure and chemical composition distribution of location A: (**a**) IPF X; (**b**) SEM picture; (**c**) Mn; (**d**) Mo; (**e**) S; (**f**) P.

In order to clarify the formation mechanism of pre-melting defects, intergranular cracks and burned holes, EBSD and EDS analysis were employed. The analysis area was region A in Figure 18a, which was also similar to region A in Figure 17a. It was found that there was a segregation of Mn, Mo, S, and P near the high-temperature crack, and the distribution trend of Mn elements was closest to the trend of the cracks.

**Figure 19:**
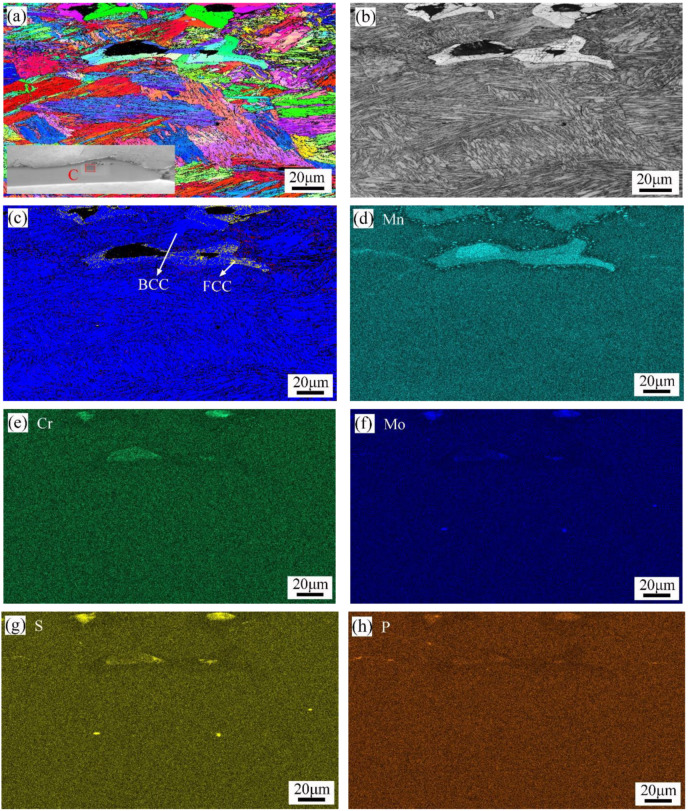
Microstructure and chemical composition distribution of location C: (**a**) IPF X; (**b**) band contrast map; (**c**) phase diagram; (**d**) Mn; (**e**) Cr; (**f**) Mo; (**g**) S; (**h**) P.

**Figure 20:**
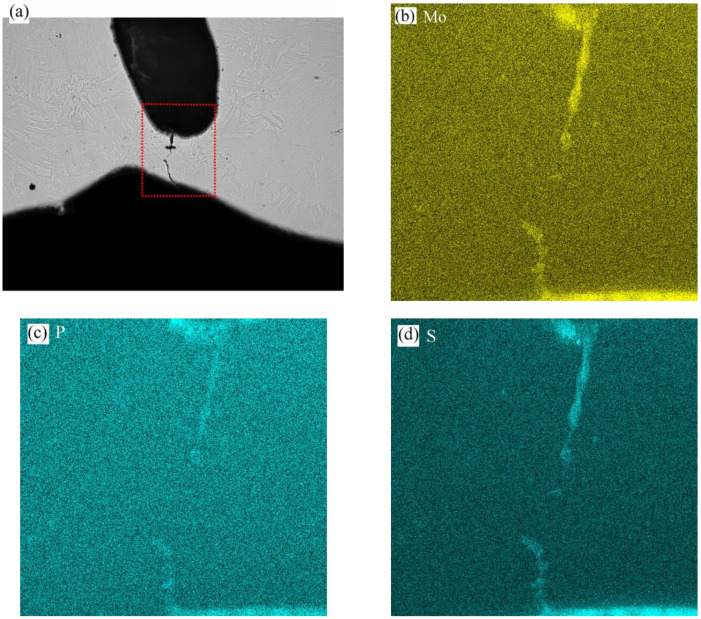
Chemical composition distribution of location B: (**a**) OM picture; (**b**) Mo; (**c**) P; (**d**) S.

Detailed EBSD, SEM, and EDS analyses were also performed in areas B and C of Figure 17a, and the results are shown in Figure 19 and Figure 20, respectively. In Figure 19, a certain amount of austenitic phase was found near the burned hole, and the apparent segregation of chemical elements such as Mn, Mo, Cr, S, and P appeared in this region. In Figure 20, the segregation of chemical elements was not very obvious, and only some of the Mo, S, and P elements were found near the cracks.

The uneven distribution of chemical elements showed an important influence on defect initiation. The chemical elements of Mn and Mo contributed to the stability of the austenite phase, and the enrichment of S and P often led to the formation of low-melting temperature compounds [38]. During the welding process, Mn elements would volatilize, and the element segregation became more serious. According to the numerical simulation, the temperature in this area was above 1300 °C, and a large number of MnS would precipitate near 1300 °C. The drastic segregation of S and P elements would lead to the formation of MnS and other compounds [39]. When MnS accumulated at the grain and subgrain boundary, it developed into a fracturing source [40]. The Mo elements were also reported to increase the risk of thermal cracking at high temperatures [41].

When the in-service welding was conducted on the pipeline with a small diameter and thin-wall thickness, the forming mechanism of the burn-through pinhole was mainly affected by the temperature, and the weld centerline crack was significantly affected by the high temperature and stress (strain) concentration. In general, burn-through instability was affected by pipeline parameters (wall thickness, pipe diameter, material), welding process (welding method, welding current, welding speed, welding voltage, et al.), and pipeline operation parameters (medium pressure, medium flow rate). Overall, the effects of key parameters on the burn-through instability obeyed the following order: wall thickness > welding heat input > medium pressure. Excessive temperature and stress (strain) concentrations were the leading causes of the burn-through instability. Burn-through instability mainly originated from the pre-melted grain boundaries and intergranular microcracks. The segregation of chemical elements, stress (strain) concentration, and high temperature facilitated the formation of the grain boundary pre-melting and the intergranular microcracks. The previous criterion was too conservative when predicting the burn-through instability risk of the pipelines with small diameters and thin-wall thicknesses. In practice, the burn-through instability would not occur until the inner wall temperature exceeded 1260 °C, while the suggested value was 982 °C for the Battelle model. According to API 1104 [7], the minimum safe wall thickness of the pipe was 6.4 mm when a low hydrogen electrode was used to conduct in-service welding. Sometimes, in-service welding must be performed on pipelines whose remaining wall thickness is less than 6.4 mm. There was no corresponding standard and experimental data to guide the in-service welding, and the burn-through instability risk was hard to evaluate. The original motivation of this research was to relax the minimum safety wall thickness limit under certain conditions and reveal the dynamic evolution behavior and mechanism of burn-through instability. Finally, the research results were expected to guide the risk assessment of thin-wall pipeline burn-through instability.

## 5. Conclusions

In this work, in-service welding on the pressurized pipe was carried out based on the designed test device, and the dynamic behaviors of burn-through instability were observed with the help of a CCD camera. After this, the failure analysis and numerical simulation were conducted to clarify the burn-through instability’s evolution behaviors and failure mechanism in detail. The main conclusions drawn were as follows.

(1). In the initial stage of burn-through instability, a large radial deformation formed beneath the molten pool bulge, and penetrating defects (pinhole or crack) appeared. The penetrating defects then quickly propagated toward the arc. Finally, the weld centerline cracks or pinholes formed.
(2). An overheating area formed during the in-service welding often developed into the initiation position of burn-through instability.
(3). Cracks and pinholes were found simultaneously in the burn-through instability zone, and both of them played an essential role in the burn-through instability. Pinholes were located in the rear of the molten pool. The weld centerline crack near the burn-through pinhole could penetrate the wall thickness.
(4). The in-service welding burn-through instability area was divided into four parts according to the failure behavior and mechanism. They were named damage failure zone (DF), burn-through instability (BTI) zone, propagation (P) zone, and secondary burn-through zone (BT2). The crack initiated from the inner surface of the DF zone and stopped in the wall thickness. The crack behavior was significantly affected by the high temperature and plastic strain. The figure-like hole and high-temperature crack were both found in the BTI zone. The failure mechanism of the BTI zone was also significantly affected by the high temperature and plastic strain. A penetrating weld centerline crack was found in the P zone, and the crack behavior was significantly affected by the DF and BTI zone. The secondary burn-through zone was mainly affected by the high temperature.
(5). There was a segregation of Mn, Cr, Mo, S, and P near the high-temperature crack, and the distribution trend of Mn elements was closest to the trend of cracks. The uneven distribution of chemical elements significantly influenced defect initiation.

## 6. Prospect

Finally, this paper reviews the original motivation of the research and prospects future research directions. The burn-through instability occurs during in-service welding and develops inside the high-temperature metal. In addition, it occurs under a very complex condition where the coupled multi-physical fields of welding interact with the pipe pressure. In essence, burn-through instability is a very complex high-temperature deformation and fracture problem. The burn-through instability behavior and mechanism were mainly discussed based on the failure analysis in previous studies. It was considered that weld centerline cracks or burn-through pinholes were the main cause of burn-through instability. However, the evolution behavior and forming mechanism of these defects were not clear, due to the lack of systematic failure analysis and in-situ observation. Hence, this research tried to clarify the above problems by combining in-situ CCD observation and failure analysis. The test devices were also developed to observe burn-through phenomena that appeared on the pipe’s outer and inner surfaces. At present, there were still many problems to be solved. Some open questions for future research are given below.

(1). Burn-through instability was affected by pipe parameters (pipe diameter, wall thickness), welding parameters (welding method, welding current, welding speed, welding voltage, et al.), and pipeline operating conditions (medium flow rate, medium pressure). The previous research mainly focused on small-diameter pipes, and the effects of the above parameters were not systematically investigated.
(2). During the internal observation, many phenomena on the inner surface of the pipeline were covered by bubbles. Hence, the crack behavior of the inner surface was difficult to observe, even with the aid of the internal observation device.
(3). Burn-through instability was affected by the multi-physics field, and in-situ observation helped clarify the behavior and mechanism. It was suggested to introduce in-situ temperature field measurement, in-situ strain field measurement, and high-speed photography to investigate burn-through instability behaviors comprehensively.
(4). There are few numerical simulation studies on the dynamic evolution process of burn-through instability. Understanding the evolution behaviors of stress, deformation, strain, temperature, and microstructure was meaningful. However, these parameters were hard to obtain and were meant to clarify the mechanism.
(5). At present, the initiation and propagation behaviors of burn-through pinholes or cracks along the wall thickness were only analyzed based on failure analysis. More evidence needs to be provided to explain the evolution of burn-through behaviors and mechanisms.
(6). The failure process of burn-through instability involved high-temperature deformation and fracture. Introducing the theory and method of fracture mechanics and damage mechanics was helpful in better understanding the failure behavior and formulating the failure criterion of burn-through instability.

## Figures and Tables

**Table 1:** Chemical compositions (in wt%) of X65.

Material	C	Si	Mn	S	P
X65	0.12	0.45	1.85	0.025	0.015

**Table 2:** The mechanical properties of X65.

Material	Tensile Strength (MPa)	Yield Strength (MPa)	Elongation (%)	Charpy Impact Energy (J)
X65	571	476	≥26.5	255/230/220

**Table 3:** Circumferential in-service welding observed with external observation set-up.

Weld Procedure	Welding Voltage (V)	Welding Current (A)	Heat Input (J/mm)	Pressure (MPa)	Evaluation
25I-1	181	14.4	456.12	6.3	Safe
25I-2	191	14.3	477.98	6.3	Safe
25I-3	206	14.2	511.91	6.3	Safe
25I-4	220	18.7	719.95	6.3	Safe
25I-5	235	16.4	674.45	6.3	Burn-through
26K-6	230	15.8	635.95	6.3	Burn-through
24C-1	200	15.4	539.0	8.5	Burn-through
24E-1	201	15.2	534.66	8.5	Burn-through
25F-1	181	15.2	481.46	9.0	Safe
25F-2	191	16.2	541.49	9.0	Burn-through
25G-1	181	16.2	513.14	9.0	Burn-through
25H-1	181	15.3	484.63	9.0	Burn-through

**Table 4:** Circumferential in-service welding observed with internal observation device.

Weld Procedure	Welding Voltage (V)	Welding Current (A)	Heat Input (J/mm)	Pressure (MPa)	Evaluation
260–1	280	14.6	740.95	3.0	Burn-through
26P-1	270	14.5	685.125	3.0	Burn-through

**Table 5:** Axial in-service welding observed with an external observation device.

Weld Procedure	Welding Voltage (V)	Welding Current (A)	Heat Input (J/mm)	Pressure (MPa)	Evaluation
17-ZD-1	160	16.2	453.6	4.5	Safe
17-ZD-2	170	16.2	481.95	4.5	Safe
17-ZD-3	180	16.2	510.3	4.5	Safe
17-ZD-4	190	16.6	551.95	4.5	Safe
17-ZD-5	200	16.6	581.0	4.5	Safe
17-ZD-6	210	16.5	606.38	4.5	Safe
17-ZD-7	220	17.2	662.2	4.5	Safe
17-ZD-8	230	17.0	684.25	4.5	Safe
17-ZD-9	245	18.0	771.75	4.5	Safe
17-ZD-10	255	18.0	803.25	4.5	Safe
17-ZD-11	270	17.8	841.05	4.5	Burn-through

**Table 6:** Circumferential in-service welding parameters.

Weld Procedure	Welding Voltage (V)	Welding Current (A)	Heat Input (J/mm)	Pressure (MPa)	Evaluation	View Angle
26K-6	230	15.8	635.95	6.3	Burn-through	External
260-1	280	14.6	740.95	3.0	Burn-through	Internal

**Table 7:** Axial in-service welding parameters.

Weld Procedure	Welding Voltage (V)	Welding Current (A)	Heat Input (J/mm)	Pressure (MPa)	Evaluation	View Angle
17-ZD-11	270	17.8	841.05	4.5	Burn-through	External

## Data Availability

The data presented in this study are available in insert article.
